# Nitric oxide-releasing gel accelerates healing in a diabetic murine splinted excisional wound model

**DOI:** 10.3389/fmed.2023.1060758

**Published:** 2023-03-02

**Authors:** Dharshan Sivaraj, Chikage Noishiki, Nina Kosaric, Harriet Kiwanuka, Hudson C. Kussie, Dominic Henn, Katharina S. Fischer, Artem A. Trotsyuk, Autumn H. Greco, Britta A. Kuehlmann, Filiberto Quintero, Melissa C. Leeolou, Maia B. Granoski, Andrew C. Hostler, William W. Hahn, Michael Januszyk, Ferid Murad, Kellen Chen, Geoffrey C. Gurtner

**Affiliations:** ^1^Division of Plastic and Reconstructive Surgery, Department of Surgery, Stanford University School of Medicine, Stanford, CA, United States; ^2^Department of Surgery, College of Medicine, University of Arizona, Tucson, AZ, United States; ^3^Center for Plastic, Reconstructive, Aesthetic and Hand Surgery, University Hospital Regensburg and Caritas Hospital St. Josef, Regensburg, Germany; ^4^Department of Biochemistry and Molecular Biology, School of Medicine, George Washington University, Washington, DC, United States

**Keywords:** nitric oxide, fibronectin, TGF-β1, wound healing, fibrosis

## Abstract

**Introduction:**

According to the American Diabetes Association (ADA), 9–12 million patients suffer from chronic ulceration each year, costing the healthcare system over USD $25 billion annually. There is a significant unmet need for new and efficacious therapies to accelerate closure of non-healing wounds. Nitric Oxide (NO) levels typically increase rapidly after skin injury in the inflammatory phase and gradually diminish as wound healing progresses. The effect of increased NO concentration on promoting re-epithelization and wound closure has yet to be described in the context of diabetic wound healing.

**Methods:**

In this study, we investigated the effects of local administration of an NO-releasing gel on excisional wound healing in diabetic mice. The excisional wounds of each mouse received either NO-releasing gel or a control phosphate-buffered saline (PBS)-releasing gel treatment twice daily until complete wound closure.

**Results:**

Topical administration of NO-gel significantly accelerated the rate of wound healing as compared with PBS-gel-treated mice during the later stages of healing. The treatment also promoted a more regenerative ECM architecture resulting in shorter, less dense, and more randomly aligned collagen fibers within the healed scars, similar to that of unwounded skin. Wound healing promoting factors fibronectin, TGF-β1, CD31, and VEGF were significantly elevated in NO vs. PBS-gel-treated wounds.

**Discussion:**

The results of this work may have important clinical implications for the management of patients with non-healing wounds.

## Introduction

Despite medical advances and various prevention efforts, diabetes mellitus has become a major global health crisis (1). More than 26 million individuals in the United States were diagnosed with diabetes in 2020, and it is estimated that an additional 90 million individuals have evidence of pre-diabetes (2). In 2017, the estimated medical cost of diagnosed diabetes was 237 billion US dollars. In addition to treatment of the disease itself, annual nonmedical costs associated with diabetes exceeded 15 billion dollars, with projections that more than 41 million individuals will be diagnosed with diabetes by 2030 (3). These figures highlight the growing financial burden this disease places on society. In addition, diabetic complications significantly reduce quality of life and diminish social productivity. Diabetic neuropathy and microangiopathy inhibit cutaneous wound closure and can result in chronic lesions, ulcers, epithelial erosion, and amputation of the extremities despite treatment efforts. Therefore, efforts to effectively promote wound healing and tissue repair of chronic wounds are increasingly relevant and vital to combat this evolving public health issue.

The mechanisms underlying wound repair involve complex biologic processes and coordinated interactions between cells, growth factors, and extracellular matrix (ECM) proteins (4). These mechanisms progress through a series of interdependent and overlapping phases including hemostasis, inflammation proliferation, and remodeling (5). Chronic wounds are wounds that have failed to progress through these ordered phases and have instead entered a state of pathologic inflammation and unresolved healing (6). The challenges associated with treating chronic wounds are potentiated by the systemic complications of diabetes, which include tissue hypoxia and decreased collagen production (7).

Nitric Oxide (NO) is an endogenous messenger molecule that plays a central role in wound healing (8). NO levels typically increase rapidly after skin injury in the inflammatory phase and gradually diminish as wound healing progresses (6). The NO molecule is produced from an oxidation process catalyzed by a group of three isozymes including endothelial nitric oxide synthetase (eNOS), inducible nitric oxide synthetase (iNOS), and neuronal nitric oxide synthetase (nNOS) (9). NO plays an important role in wound healing by mediating vascular hemostasis, inflammation, and antimicrobial action. Decreased production of NO is characteristic of diabetes and has been associated with impaired healing in chronic wounds. Studies have shown that the topical application of NO-releasing agents on wounds can stimulate cell proliferation, increase the production of collagen and growth factors, and accelerate angiogenesis (10–12). However, the effect of increased NO concentration in the context of diabetic wound healing has yet to be described. In this study, we investigated the effect of local administration of an NO-releasing gel on excisional wound healing in diabetic mice.

## Materials and methods

### Animals

Genetically diabetic db/db mice (BKS.Cg-m 1/1 Leprdb/J) were obtained from Jackson Laboratories (Bar Harbor, ME) (strain #: 697). These homozygous db/db mice possess a genetic mutation of the leptin receptor and represent a model of type 2 diabetes characterized by impaired wound healing, obesity, hyperglycemia, and hyperinsulinemia (Supplementary Table 1). Animal care was provided in accordance with the Stanford University School of Medicine guidelines and policies for the use of laboratory animals.

### *In vivo* stented excision wound model

Female db/db mice were randomized into two treatment groups: NO-gel or PBS-gel control (n = 5 mice per group). Splinted full-thickness excisional wounds were created as previously described by Galiano et al. (13) A full-thickness wound was excised using a sterile 6-mm punch biopsy tool on each side of the dorsal midline. Each wound was splinted with donut-shaped silicone splints cut from a 0.5 mm silicone sheet (Grace Bio-Laboratories, Bend, OR). The splint was centered around the wound, affixed to the skin using a bonding adhesive (surgical glue), and then sutured in place to prevent wound contracture and promote granulation tissue formation to mimic human wound healing. Two excisional wounds of each mouse received either NO-gel or control PBS-gel treatment twice daily until complete wound closure. All wounds were covered with a sterile occlusive dressing (Tegaderm, 3 M, St. Paul, MN, United States). Wound dressings were changed once per day for the duration of the experiment. Digital photographs were taken on day 0 and 1 and every other day thereafter until complete wound closure. The wound areas were quantified using ImageJ and expressed as a percentage of the original wound area.

### NO-gel preparation and application

Based on Zhu’s method, a warm solution of sodium nitrite (14.6 mM) in distilled water was introduced into a gel by adding hydroxyethyl cellulose (molecular weight 50,000–1,250,000) (9, 14).

This dosage was chosen as it releases a comparatively constant maximal output of NO over time. In the current study, 2 g sodium nitrite was dissolved in 100 mL 3.2 g/100 mL cellulose solution to prepare nitrite gel, and 0.85 g maleic acid and 1.3 g vitamin C were dissolved in 25 ml 3.2 g/100 mL cellulose solution to prepare low pH acid gel. After mixing equal amounts of the two gels immediately before use, the mixture was placed on the excisional wound. This dosage for application was established based on an approved protocol by the National Institute of Health-Small Business Technology Transfer (NIH-STTR) grant. The release kinetics of this nitric oxide gel has previously been monitored by an amperometric electrode technique (amiNO-2000 NO Sensor, Innovative Instruments, In. Tampa, FL). This NO-release study showed that the concentration of NO can be maintained at 10 nM within the wound bed over 1 h after application (9). The PBS gel was prepared by exchanging sodium nitrite for sodium phosphate. The sodium nitrite and low pH gel prepared with the addition of maleic acid and ascorbic acid were mixed prior to application, and subsequently applied to the wound area, covering the area entirely. Immediately after wounding, either NO-releasing gel (1 × 10 in 100 μL of PBS) or the same volume of PBS-gel was applied onto wound twice daily until wound closure, and the rate of wound healing was evaluated every other day.

### Histological analysis of collagen content and architecture

Wounds were harvested on day 2 and 7 after wounding, and healed scar tissue was harvested at the end of the study on day 21. Tissues were fixed in 4% paraformaldehyde overnight, dehydrated with sequential ethanol concentrations (30%, 50%, 70%, and 95%), xylene, and paraffin washes, and embedded in paraffin for sectioning. Hematoxylin and Eosin (H&E) and Masson’s Trichrome staining were performed according to the manufacturer’s recommendations, and images were captured with a Leica Aperio AT2 digital whole slide scanner. We implemented an algorithm in MATLAB to automatically deconvolute the color information of each Trichrome image (15). This algorithm allows for a robust and flexible method for objective immunohistochemical analysis of samples stained with up to three different colors. Picrosirius Red (Sigma Aldrich) staining was also performed, and we utilized a Leica DM5000 B upright microscope for linear polarized light microscopy to capture images of the Picrosirius Red-stained images. Polarized light was oriented to maximally display fibers parallel to the skin surface. Collagen fiber quantification was performed using CT-FIRE and CurveAlign, an open-source software package for automatic segmentation and quantification of individual collagen fibers^1^ (15). Briefly, CurveAlign quantifies all fiber angles and the strength of alignment within an image, while CT-FIRE analyzes individual fiber metrics such as length, width, angle, and curvature. The average fiber parameters for each mouse were used for statistical analysis. Finally, complexity and heterogeneity were measured using the ImageJ plug-in FracLac (16). The software analyzes tissue morphology using fractional dimensions to determine the lacunarity (L) values using the subsample box counting scan (50 grid default sampling size, minimal pixel density threshold = 0, and rectangle subscan). L measures the amount of randomness or heterogeneity in a sample. A low L implies less heterogeneous collagen fiber orientation.

### Immunofluorescent staining

Immunofluorescent staining was performed using primary antibodies Fibronectin (1:100 dilution, Abcam, Ab2413), TGFβ1 (1:100 dilution, Abcam, Ab215715), VEGF (1:100 dilution, Thermo Fisher Scientific, PA1-21796), and CD31 (Abcam, ab28364). The percentage of fluorescent area was quantified using a custom MATLAB image processing code written by the authors and previously published (17). All immunofluorescent images shown are representative images.

### Statistical analysis

Statistical analysis was performed in Prism8 (GraphPad, San Diego, California). Continuous variables were assessed using an unpaired Student’s t-test or two-way analysis of variance (ANOVA). Data were presented as means ± standard error of the mean. Sample sizes (n) and *p* values are indicated in the figure legends. Values of *p < 0.05 were considered statistically significant.

## Results

### Nitric oxide-releasing gel accelerates excisional wound healing in db/db mice

**Figure 1:**
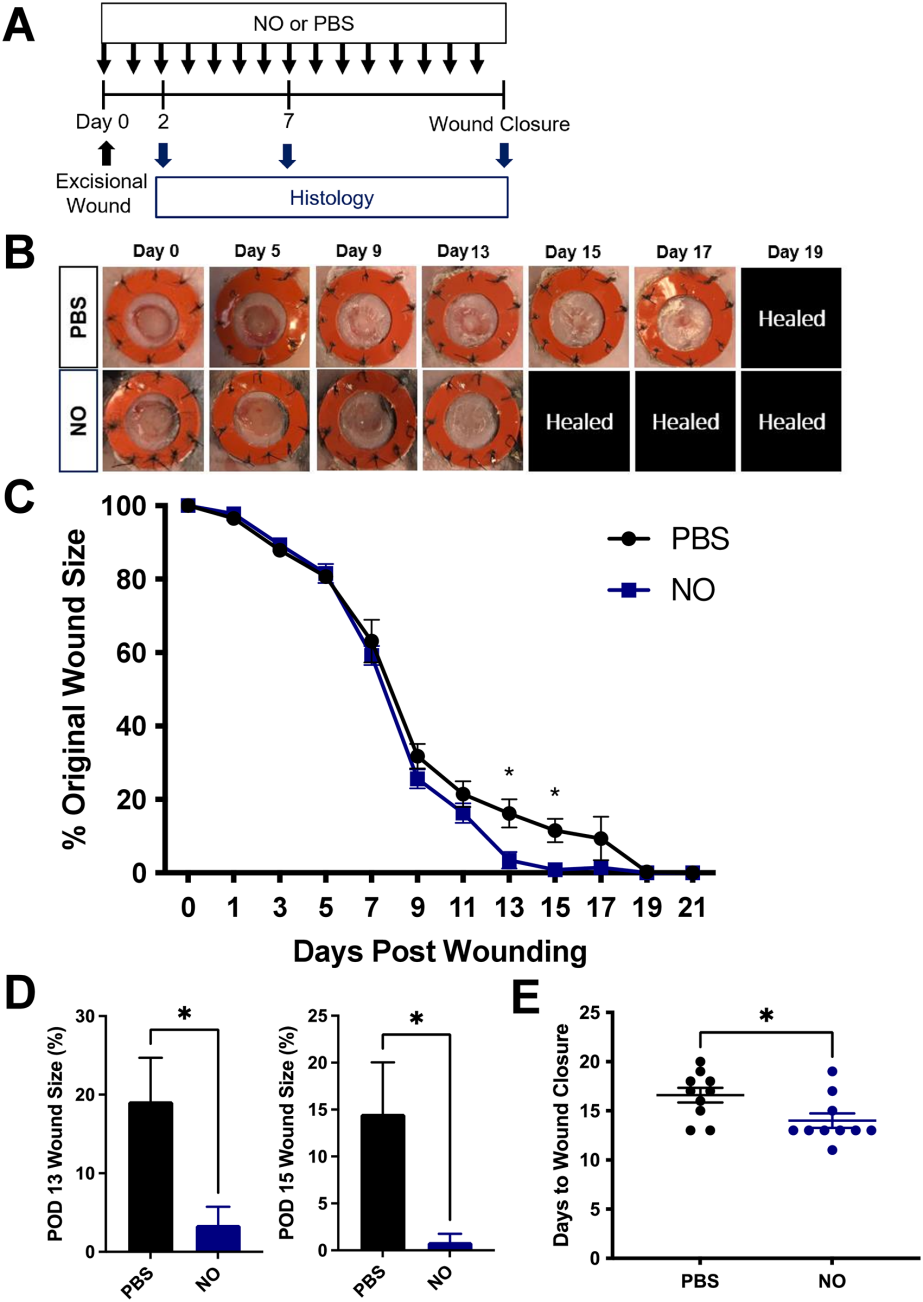
**(A)** Experimental overview of excisional wounding and treatment. **(B)** Representative images of the wound over time by treatment group, where NO = Nitric Oxide gel; PBS: Phosphate-Buffered Saline-gel control. Healed = healed wound that has closed. **(C)** Quantification of wound area over time by treatment group. **(D)** Wound area size at postoperative (POD) 13 and 15. **(E)** Days until complete wound closure by treatment group. Data are presented as mean value ± SEM, *p < 0.05.

The efficacy of topical administrated NO-releasing gel on wound healing was evaluated in a mouse excisional wound healing model as described previously (Figure 1A). To measure the effect of each hydrogel treatment on wound healing, we assessed wound area change over time by analyzing digital photographs that were taken during each dressing change (Figures 1B,C). The wound size is represented as an average size of 10 wounds per treatment group (5 mice per group, 2 wounds per mouse). At postoperative days 13 and 15, wounds treated with the NO gel were significantly smaller than wounds treated with PBS (Figure 1C). The absolute wound percentage sizes are also shown as individual bar graphs (Figure 1D) to further demonstrate this significant difference at both postoperative day (POD) 13 (*p < 0.05) and POD 15 (*p < 0.05). We then assessed the digital photographs of each mouse wound to determine the average number of days before complete wound closure for each treatment group. The mean time for complete wound healing was 14.0 ± 0.75 days in the NO-gel-treated group, significantly faster than 16.0 ± 0.75 days in the PBS-gel-treated group (*p < 0.05, Figure 1E).

### Nitric oxide-releasing gel improves collagen architecture in healed diabetic wounds

**Figure 2:**
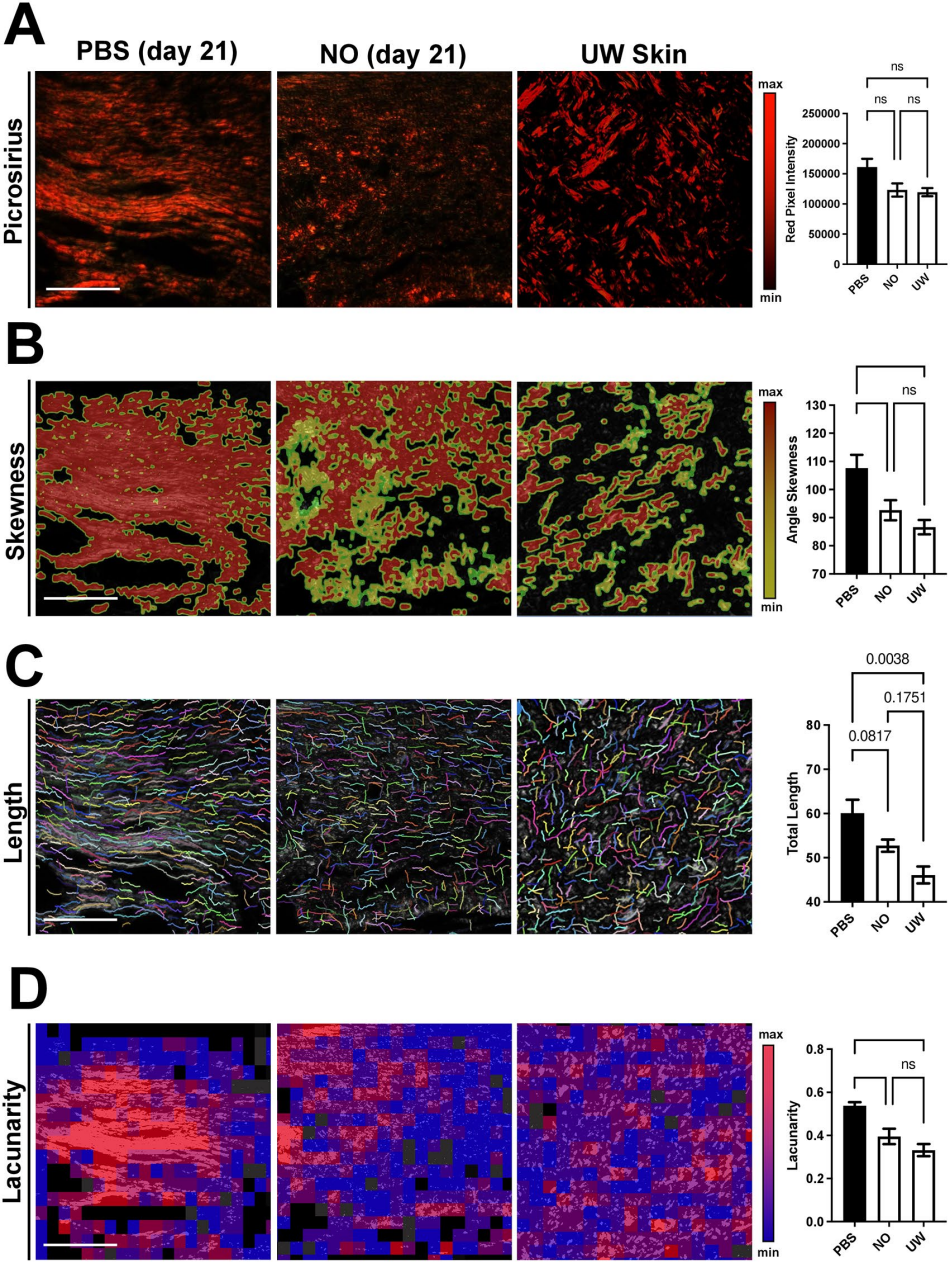
Picrosirius red staining and comparison of NO and PBS-gel-treated wounds, using collagen algorithms CurveAlign, CT-Fire, and FracLac. Scale bars: 200 μm. Quantification of **(A)** collagen fiber pixel intensity, **(B)** fiber angle skewness, **(C)** fiber length, and **(D)** tissue lacunarity. ns = nonsignificant. Data are presented as mean value ± SEM, *p < 0.05.

Collagen tissue quality of healed wounds in the NO- and PBS-gel-treated groups at day 21 was evaluated using picrosirius red staining, which highlights collagen networks by making use of the birefringent properties of collagen molecules, to evaluate the collagen density and orientation of the scars in each group. Analysis of unwounded (UW) skin was included for comparison. The red pixel intensity among PBS and NO-gel-treated healed scars and UW skin were similar, indicating a comparable amount of mature collagen within the healed scars in both groups (Figure 2A). A quantitative assessment of the collagen architecture of the wounds was then performed using the software algorithms CT-Fire, CurveAlign, and FracLac, which have been previously developed for analysis of collagen fiber properties on histology images (18–20). We utilized this array of metrics to analyze the fiber length, angle skewness, red pixel intensity, and fiber lacunarity of the tissues. Using CurveAlign, we found that NO-gel-treated wounds showed significantly more random alignment compared to PBS-gel-treated wounds and displayed a similar phenotype to that of UW skin (*p < 0.05; Figure 2B). Using CT-FIRE, we found that NO-gel-treated wounds and UW skin also demonstrated a trend toward shorter fiber lengths compared to PBS-gel-treated wounds (*p* = 0.0807; Figure 2C). Finally, using FracLac analysis to assess the complexity and heterogeneity of the healed scars in all groups, we found that NO-gel-treated wounds and UW skin displayed significantly greater lacunarity compared to PBS-gel-treated wounds (*p < 0.05), indicating a more heterogeneous collagen fiber network orientation (Figure 2D). Lacunarity measures the number of gaps in the tissue and thus is a surrogate marker of tissue density. We found that NO-gel-treated wounds had a porous architecture akin to that of UW skin. Taken together, our results suggest that NO-gel promoted shorter and more randomly aligned collagen in the wound bed, more like the collagen fiber networks present in UW skin (Figures 2A–D) (4, 18, 21, 22). In contrast, PBS-gel-treated wounds promoted a densely aligned collagen network with long fibers typically associated with fibrotic tissue.

### Nitric oxide-releasing gel improves dermal structure in healed diabetic wounds

**Figure 3:**
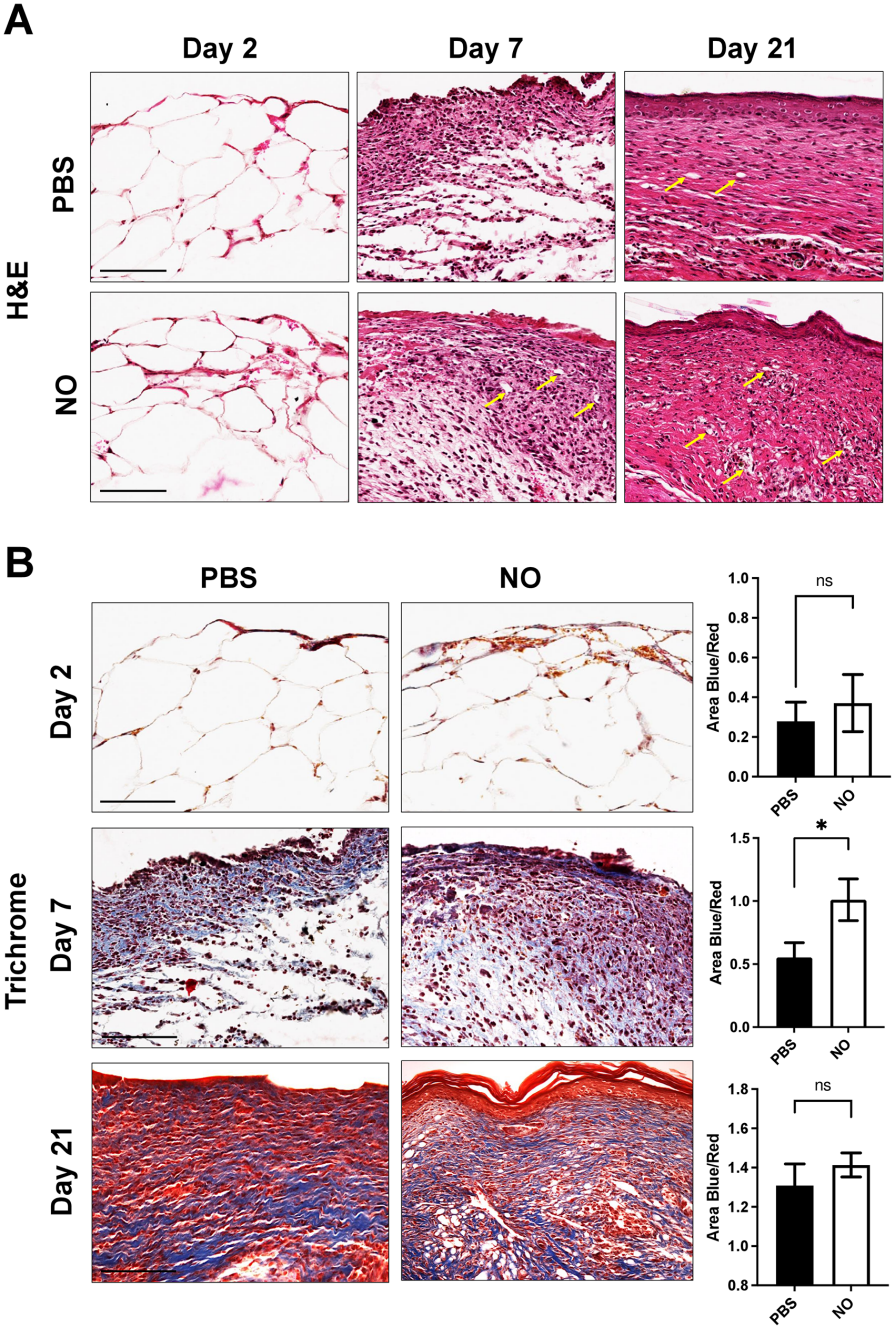
**(A)** Representative H&E images of tissue sections on days 2, 7, and 21 (healed) showing cells (nuclei in purple) and extracellular matrix (pink) in all groups. Arrows indicate blood vessels. Scale bars: 150 μm. **(B)** Masson’s trichrome staining of representative tissue sections showing dermal structure of NO and PBS-gel-treated wounds on days 2, 7, and 21 (healed). Analysis for total area positive for collagen (area blue). Scale Bar: 200 μm.

The tissue composition of murine scar tissue was qualitatively assessed using Hematoxylin and Eosin (H&E) staining, which showed, on average, increased cellularity in the NO-gel-treated scars compared to the PBS-gel-treated scars (Figure 3A). Dermal structure of murine scar tissue was assessed using Masson’s Trichrome staining (Figure 3B). Trichrome staining confirmed the picrosirius red staining analysis results, showing a more randomly aligned collagen fiber network in the NO-gel-treated healed scars on day 21. In contrast, PBS-gel-treated healed scars on day 21 were characterized by longer and more avascular bundles of collagen. The collagen area was similar and nonsignificant between the NO and PBS-gel-treated groups. On day 2 of treatment, there were minimal differences in collagen deposition and area between the two groups. Interestingly, on day 7 of treatment, there was significantly higher collagen deposition in the NO-gel-treated wounds (Figure 3B).

### Nitric oxide-releasing gel increases expression of wound healing promoting factors

**Figure 4:**
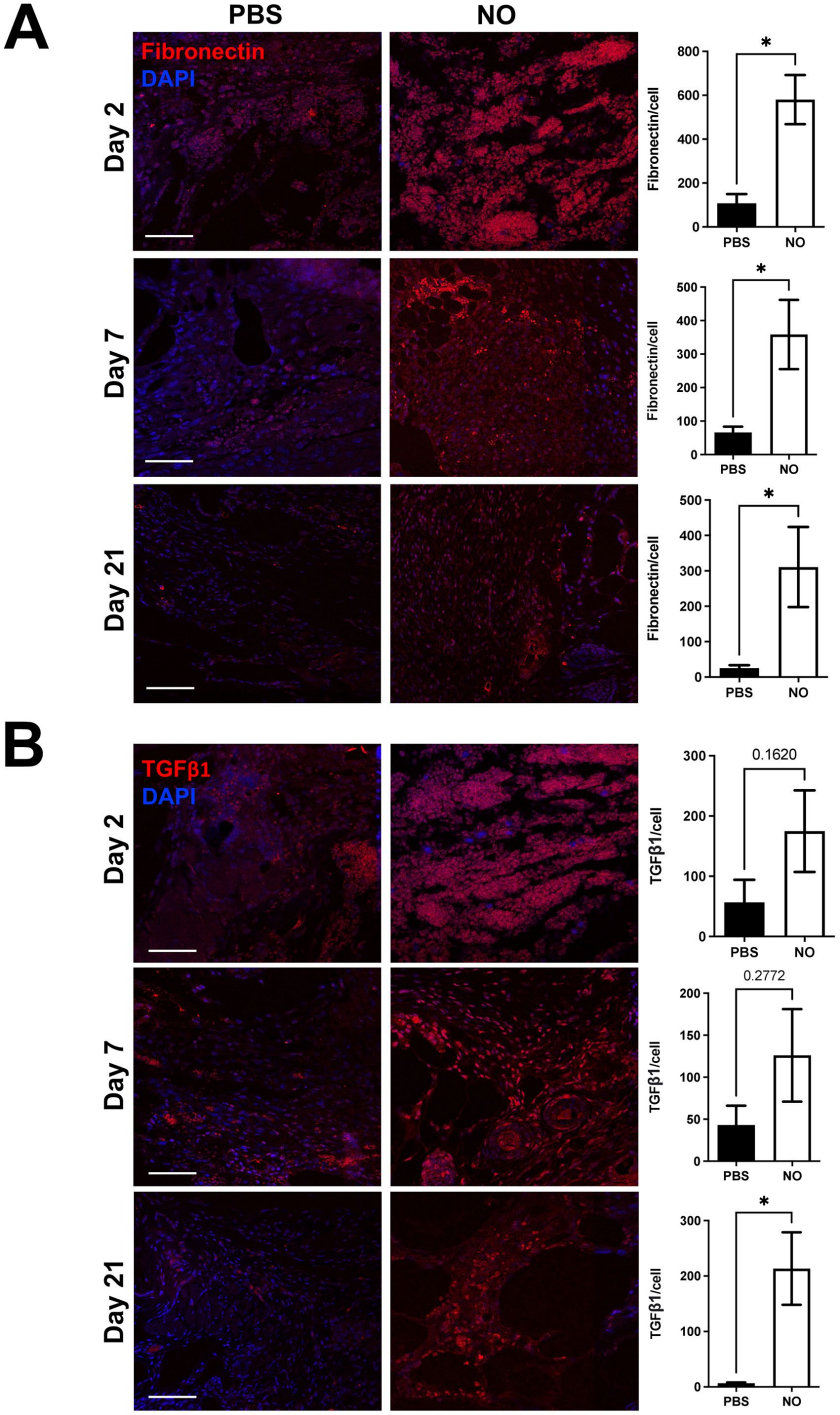
**(A)** Immunostaining for Fibronectin and **(B)** TGF-β1 in tissue sections on days 2, 7, and 21 (healed). Scale bars: 50 μm. Quantification of percent area positive for marker in each section. Data are presented as mean value ± SEM, *p < 0.05.

To assess the effect of NO on wound healing promoting factors, we performed immunostaining of fibronectin and TGF-β1, which have been shown to be reduced in abnormal wound repair and in chronic wounds (23–28). First, we observed that expression of fibronectin was significantly higher at days 2, 7, and 21 (post healing) in the NO-gel-treated group compared to the PBS-gel-treated group (Figure 4A). Further, fibronectin levels appeared to be consistently maintained over time in the NO-gel-treated group, while levels appeared to decrease over time in the PBS-gel-treated group. We observed that expression of TGF-β1 progressively decreased over the course of PBS-gel treatment and was significantly lower than in the NO-gel treatment group on day 21 (*p < 0.05) (Figure 4B). Staining for markers of angiogenesis, CD31 and VEGF, in explanted scar tissue revealed significantly higher expression of both markers in the NO-gel-treated group compared to the PBS-gel -treated group (*p < 0.05) (Supplementary Figure S1). Taken together, these results suggest that NO-gel treatment is associated with a cascade of downstream effects, including upregulation and sustained maintenance of wound healing and angiogenic promoting factors. Thus, administration of exogenous NO promotes a healing phenotype that reverses the impaired wound healing observed in diabetic mice.

## Discussion

Previous studies have shown that topical NO-releasing agents enhance excisional wound healing in diabetic models *via* a variety of mechanisms including increased cell infiltration, cytokine release, and growth factor production (6, 8, 10–12, 14). However, the tissue architectural changes in collagen structure and alignment resulting from the application of exogenous NO in diabetic wound healing have not been described. Here, we found that the application of NO-gel treatment accelerates wound healing and promotes tissue with shorter, less dense, and more randomly aligned collagen fibers, more similar to the natural architecture of unwounded skin. Further, we show that application of NO-gel treatment elevates expression of fibronectin and TGF-β1 throughout the healing process, as well as elevates expression of angiogenic factors CD31 and VEGF within the healed tissue.

We found that the NO-gel-treated and PBS-gel-treated diabetic wounds had similar rates of wound closure until approximately day 9, when the NO-gel-treated wounds began to close more rapidly. This divergence indicates that our treatment produces the most significant effects toward the later stages of diabetic wound healing. Interestingly, although the total collagen area in both groups was similar in the healed scars by day 21, the resultant tissue architecture was markedly different between the two groups. Our unbiased collagen analysis showed that healed tissue from NO-gel-treated wounds exhibited a “basket weave”-like collagen fiber network, resembling the physiologic dermal collagen architecture of unwounded murine skin. This contrasted with PBS-gel-treated wounds, which were predominantly composed of large, long bundles of avascular collagen and a less robust tissue architecture. A “basket weave”-like tissue architecture has been associated with significantly higher resistance to mechanical tensile forces compared to scars that display more highly aligned collagen networks (29–31). Our immunohistochemical analyses showed that levels of fibronectin and TGF-β1 progressively decreased in the PBS-gel-treated group but remained persistently elevated in the NO-gel-treated group over the course of healing.

Fibronectin is a large glycoprotein that provides critical linkage between the ECM and integrins (24). During healing, fibronectin acts as a building block that helps to facilitate the formation of more mature ECM (e.g., collagens), granulation tissue, and new epithelial tissue in concert with fibroblasts and other cell types (23). Reduced fibronectin matrix deposition is associated with chronic wound healing and an inability to form a new ECM in the wound bed (32–34). NO-synthase has been shown to be directly involved in enhancing fibronectin production by endothelial cells (35). Upregulated fibronectin expression was observed as early as day 2 and then persistently throughout all time points, which likely helped to promote accelerated wound closure, ECM reconstruction, and overall beneficial tissue healing.

Chronic wounds, including diabetic foot ulcers, have been found to exhibit a lack of expression of all transforming growth factor (TGF-β) isoforms (27). Specifically, fibroblasts from diabetic wounds, which are recruited to the wounds from immune cells, appear to exhibit impaired TGF-β signaling and decreased ECM synthesis (26, 36). In the context of wound healing, TGF-β is involved in angiogenesis, fibrosis, as well as the production and maintenance of ECM components including fibronectin and collagen (26). TGF-β downregulates the expression and activity of matrix-degrading enzymes such as MMPs, which are highly upregulated in diabetic wounds. Some studies have suggested a mutual feedback mechanism between nitric oxide synthase (NOS) and TGF-β1 where NOS may be exerting its action within the wound bed *via* signaling of TGF-β1, leading to fibroblast activation and collagen production (25, 28, 37–40). In normal wound healing, TGF-β1 secreted from macrophages stimulates granulation tissue formation, collagen formation, and ECM remodeling (5). Our data indicate that NO-gel treatment is associated with steadily increasing TGF-β1 levels within the wound bed, which is likely linked in part to the improved tissue quality we observed.

Overall, our findings suggest that NO-gel treatment in chronic diabetic wounds accelerates wound healing and promotes a scar phenotype more similar to the natural basket-weave architecture of unwounded skin. The direct and indirect effects of NO pharmacologically accelerate wound healing, likely in part, by increasing angiogenesis and production of fibronectin and TGF-β1 within the wound bed. These factors lay the appropriate foundation for normal ECM reconstruction, angiogenesis, and tissue reconstruction in chronically impaired wounds. We show that by restoring the physiological environment present in normal wound healing, we can promote tissue reconstruction and accelerate healing in diabetic wounds. Future studies will need to be performed to interrogate the molecular mechanisms driving healing from exogenous NO therapy, as well as the relationship between NO, fibronectin, TGF-β1, and angiogenesis in chronic wound healing.

## Data availability statement

The original contributions presented in the study are included in the article/Supplementary material, further inquiries can be directed to the corresponding authors.

## Ethics statement

The animal study was reviewed and approved by Stanford University School of Medicine.

## Author contributions

NK, HaK, CN, DS, KC, FM, and GG designed the study. HaK, NK, HuK, KF, DH, AT, BK, ML, MG, AH, and WH performed animal experiments and data analysis. DS, CN, and NK wrote the manuscript. GG and KC helped to revise and edit the manuscript. All authors contributed to the article and approved the submitted version.

## Conflict of interest

The authors declare that the research was conducted in the absence of any commercial or financial relationships that could be construed as a potential conflict of interest.

## Publisher’s note

All claims expressed in this article are solely those of the authors and do not necessarily represent those of their affiliated organizations, or those of the publisher, the editors and the reviewers. Any product that may be evaluated in this article, or claim that may be made by its manufacturer, is not guaranteed or endorsed by the publisher.

## Footnotes

^1^ http://loci.wisc.edu/software/ctfire

## Abbreviations

FN: Fibronectin
TGF-β1: Transforming growth factor beta-1
NO: Nitric oxide
