# Supplementary figures and images for: Nitric oxide-releasing gel accelerates healing in a diabetic murine splinted excisional wound model
Source: Front Med (Lausanne). 2023 Mar 2;10:1060758. doi: 10.3389/fmed.2023.1060758 (PMC10045479; doi:10.3389/fmed.2023.1060758)

# Supplementary Figure S1

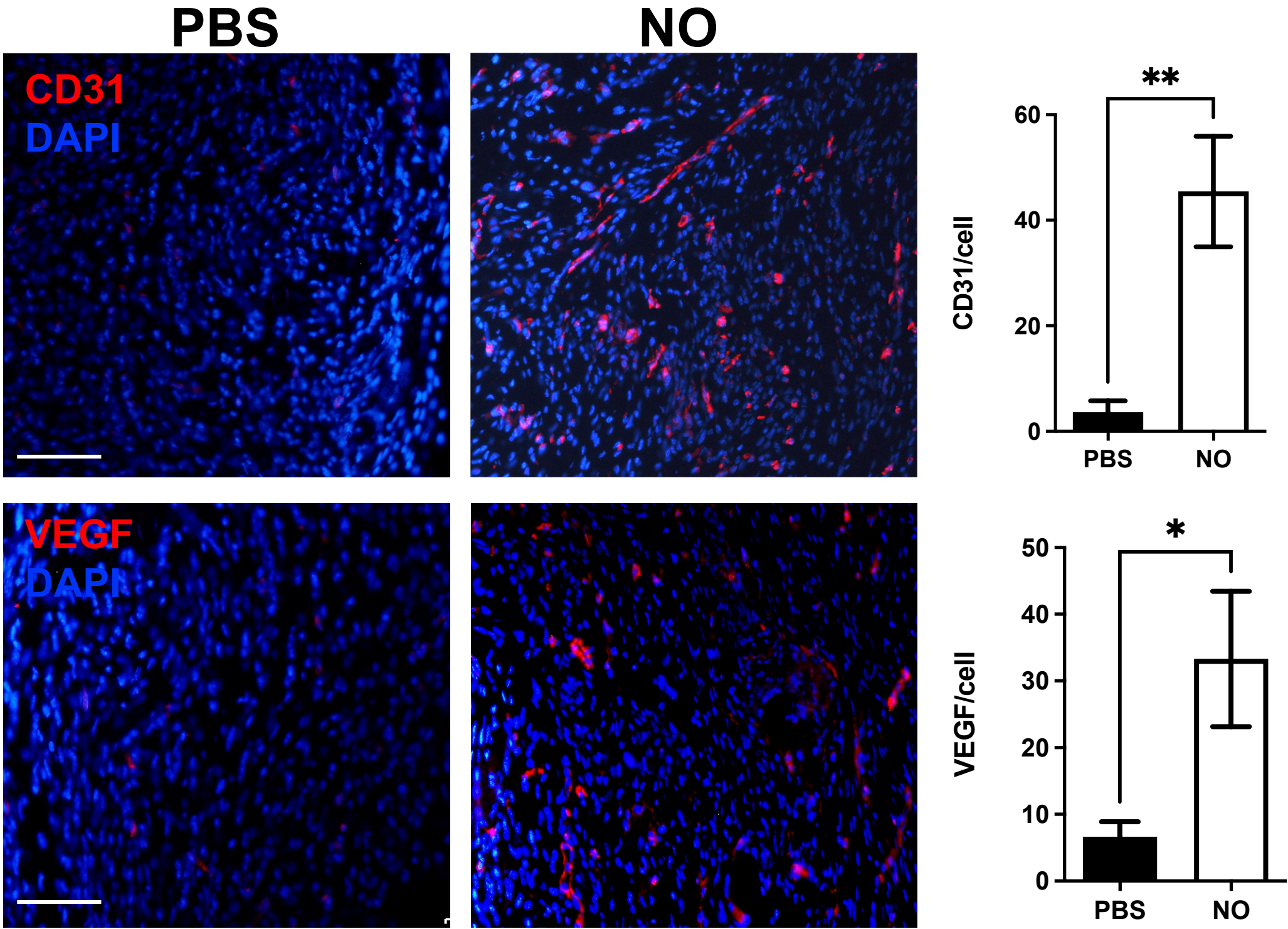

Supplement: Supplementary file 1 [file Image_1.pdf]
