# Supplementary material for: Nitric oxide-releasing gel accelerates healing in a diabetic murine splinted excisional wound model
Source: Front Med (Lausanne). 2023 Mar 2;10:1060758. doi: 10.3389/fmed.2023.1060758 (PMC10045479; doi:10.3389/fmed.2023.1060758)
Supplement: Supplementary file 2 [file Table_1.pdf]

Supplementary Table 1

Mouse	Glucose level (day 0)	Mouse	Glucose level (day 0)
PBS-1	252 (mg/dL)	NO-1	333 (mg/dL)
PBS-2	302 (mg/dL)	NO-2	313 (mg/dL)
PBS-3	289 (mg/dL)	NO-3	244 (mg/dL)
PBS-4	304 (mg/dL)	NO-4	287 (mg/dL)
PBS-5	389 (mg/dL)	NO-5	324 (mg/dL)
